# Open Reduction and Internal Fixation of a Proximal Femoral Shaft Fracture in a Patient with Bilateral Congenital Hip Disease

**DOI:** 10.1155/2018/2070564

**Published:** 2018-01-31

**Authors:** Stefania Kanata, Antonios Anastasiadis

**Affiliations:** 6th Orthopaedic Department, KAT General Hospital of Athens, Nikis 2, Kifissia, Athens 145-61, Greece

## Abstract

We present a rare case of a femoral shaft fracture in a 74-year-old woman with a preexisting untreated bilateral congenital hip dislocation and with concomitant leg length discrepancy. Because of the marked deformity of the upper femur, a derivative of the congenital hip disease, an open reduction and internal fixation was performed, with the use of an anatomic femur plate originally designed for the treatment of periprosthetic fractures. The patient was treated successfully and returned to her previous status of activity. The management of a femoral fracture in the presence of ipsilateral hip disease is a challenging issue. The surgical treatment choice in such cases has to be individualized, taking into consideration several anatomic and medical parameters.

## 1. Introduction

Congenital hip disease (CHD) is relatively common in Greek people [1]. The incidence of untreated cases has been effectively reduced, due to screening methods and early diagnosis. However, still there is sporadic presence of neglected CHD cases, particularly in elders. The occurrence of a femoral shaft fracture in the presence of CHD is extremely rare. Therefore, there are no treatment guidelines for such cases. Intramedullary nailing, the method of choice for the treatment of most femoral shaft fractures, cannot be easily performed in patients with concurrent ipsilateral CHD, especially in those with the severe type of it.

We present here a case of a femoral shaft fracture in a female patient with untreated bilateral congenital hip dislocation. A good final outcome was achieved with internal fixation of the fracture using a periprosthetic fracture plate.

## 2. Case Presentation

**Figure 1:**
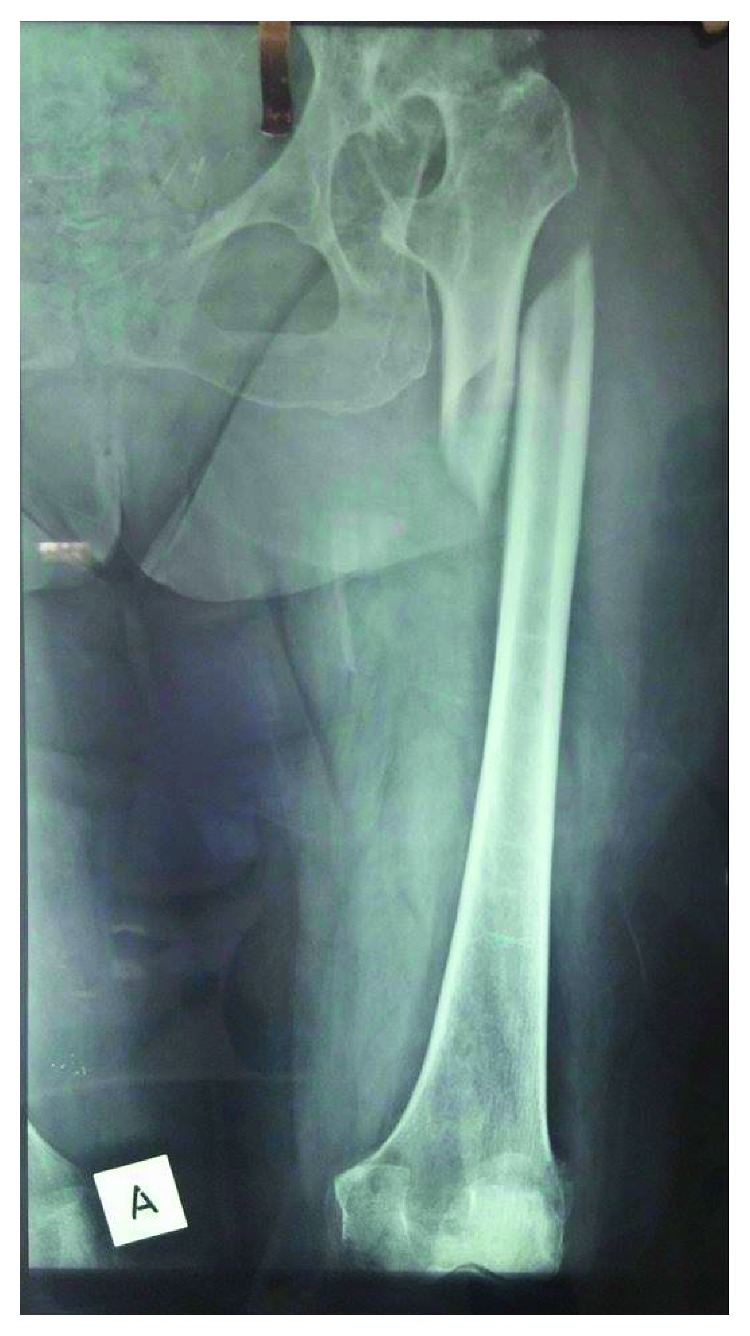
Initial posttraumatic anteroposterior radiograph of the left femur. Congenital hip disease and spiral shaft fracture of the proximal third of the femur are revealed.

**Figure 2:**
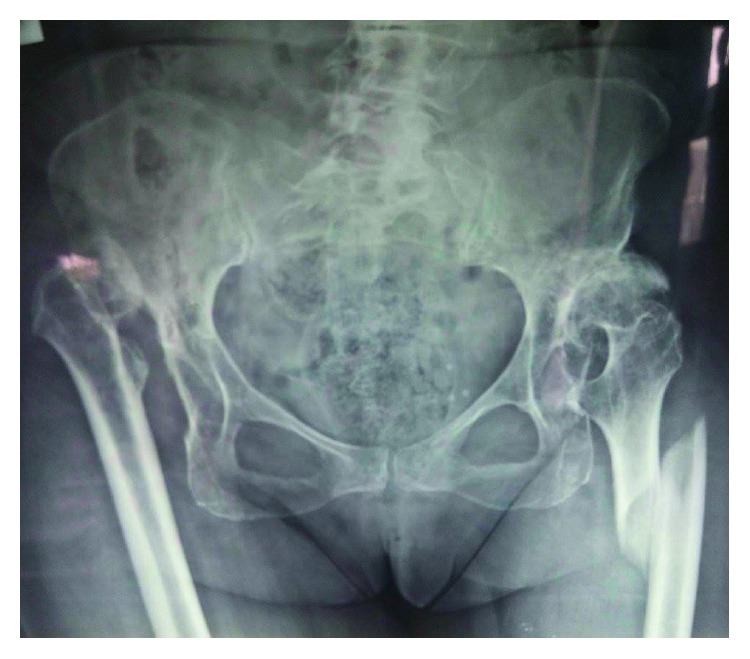
Pelvis anteroposterior radiograph showing the neglected hip dislocation of both hips.

A 74-year-old woman was admitted to the emergency department of our hospital after a closed injury of her left femur due to a fall. Plain X-rays showed a spiral shaft fracture of the proximal third of the femur. The radiographs of the pelvis and the patient's history as well revealed that she was also suffering from bilateral congenital hip disease (Figures 1 and 2). The form of dysplasia of the left hip was type B2-low dislocation according to Hartofilakidis classification or type III according to Crowe classification [2–4]. The right hip lesion was more severe, classified as type C2-high dislocation or type IV according to Hartofilakidis and Crowe classifications, respectively [2–4]. There was a leg length discrepancy of 3 cm, with the injured leg being the longer one. Before the fracture, the left hip was almost immovable, ankylosed in fixed external rotation. The right hip was free of pain with functional range of movement. The patient had never been treated for the CHD, and she was walking with the use of a cane. She was also able to do the usual activities of daily living without significant difficulty. According to her medical history, she was a smoker and was receiving medication for arterial hypertension and hypothyroidism.

**Figure 3:**
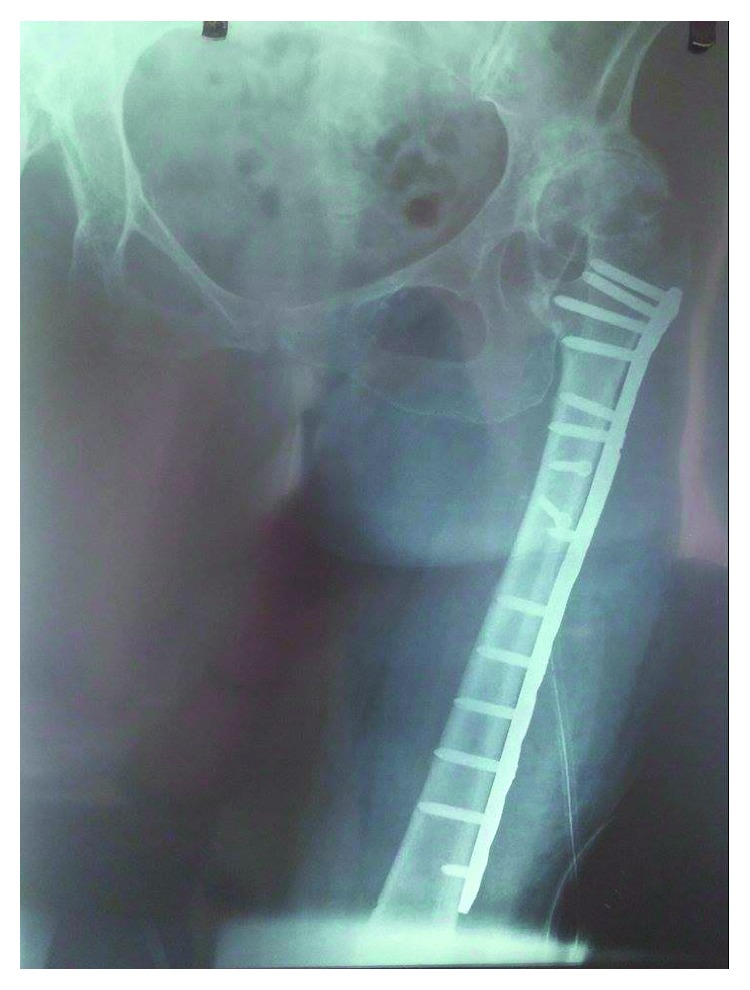
Anteroposterior radiograph of the left femur after ORIF (POD 1).

Taking into consideration the “personality” of the fracture, the patient's needs, and the surgical options, we performed an open reduction and internal fixation (ORIF) using the NCB periprosthetic femur polyaxial locking plate system (Zimmer Biomet, Indiana, USA). The operation was performed two days after the fracture, under spinal anaesthesia. The patient was placed in the right lateral decubitus position, and the middle and proximal thirds of the femur were accessed through a lateral approach. The subtrochanteric area of the femur was exposed with anterior retraction of vastus lateralis after an L-shaped detachment of its origin. The fracture was anatomically reduced, and two lag screws were placed initially. Then, the NCB periprosthetic femur plate was placed as a neutralization plate (Figure 3). Image intensifier was used for the confirmation of the optimal position of the plate. The patient received the standard scheme of perioperative chemoprophylaxis with teicoplanin as well as pre- and postoperative thromboprophylaxis with the use of bemiparin sodium. Intraoperatively, she was transfused with two units of concentrated red blood cells.

**Figure 4:**
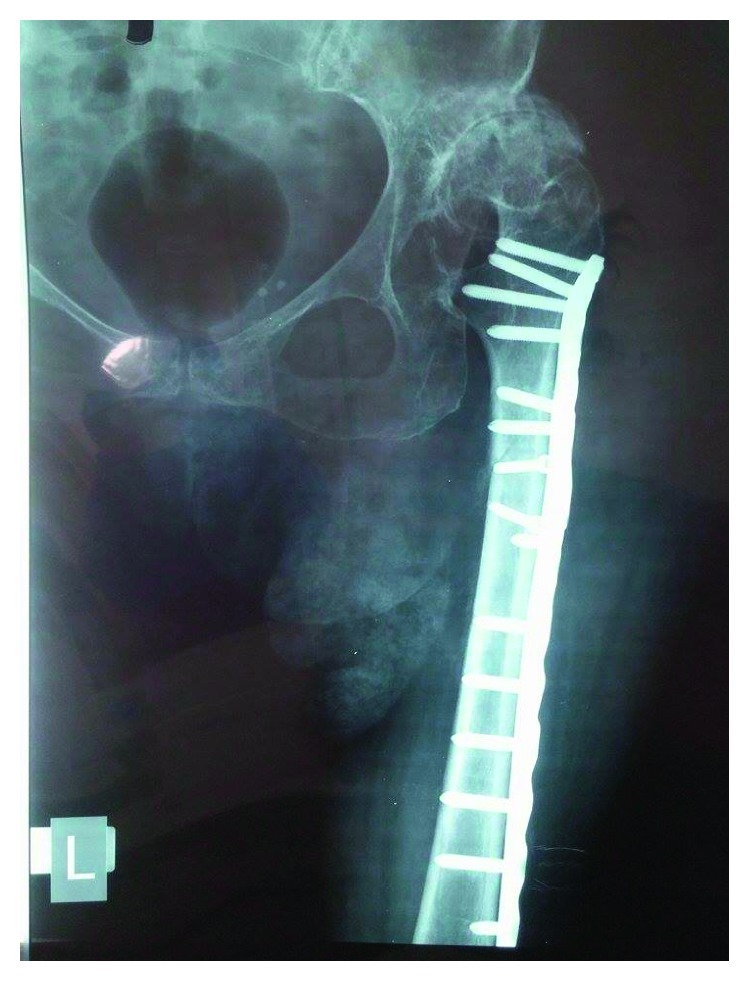
Anteroposterior radiograph of the left femur six weeks postoperatively.

**Figure 5:**
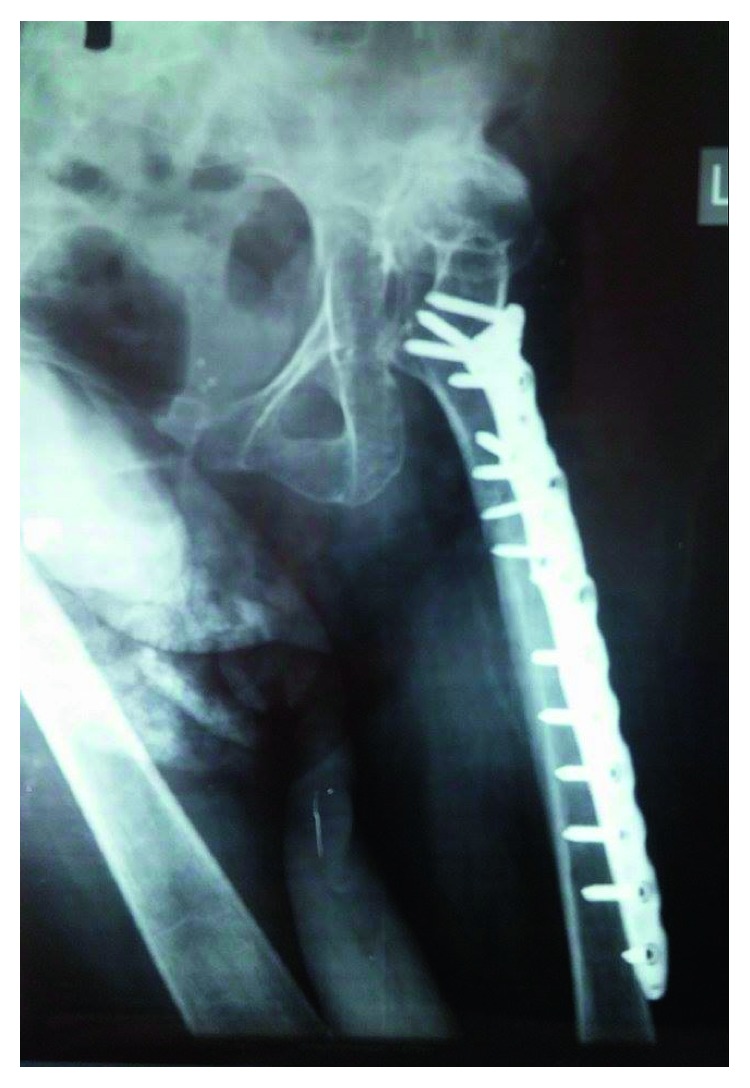
Profile radiograph of the left femur six weeks postoperatively.

The patient was mobilized on the first postoperative day (POD), and she stood upright using crutches, without weight bearing on the fourth POD. The postoperative period was uneventful, and she was discharged on the 6th POD. Because of the leg length discrepancy, she was protected from weight bearing for six weeks when she was reviewed in our outpatient clinic. At that time, the patient was free of pain, and the subsequent X-ray images showed signs of fracture healing (Figures 4 and 5). Following that, she was encouraged to start walking with partial weight bearing. The patient is being followed up for ten months now, and she has fully returned to her previous walking status and activities of daily living.

## 3. Discussion

The combination of a femoral fracture in the presence of preexisting hip pathology is extremely rare. Most of the published reports refer to the cases of either the upper femur or femoral shaft fracture under an arthrodesed hip [5–10]. To the best of our knowledge, there is only one case in the literature concerning the femoral fracture in a preexisting CHD [11].

In our case, it was a noncomminuted spiral fracture of the femoral shaft, caused by rotational forces after a simple fall. The gold standard for those fractures is closed intramedullary nailing (IMN) [12]. However, in patients with CHD, there are alterations in anatomy, increased anteversion (varying from 1 to 80 degrees), increased neck-shaft angle, and hip ankylosis, which make IMN a method with great difficulties and potential intraoperative obstacles and risks [13]. The increased neck-shaft angle combined with the increased anteversion also exclude the fixation with dynamic hip screw (DHS) or other angled plates. Reconstruction with total hip arthroplasty (THA) with long femoral stem was an option thoroughly considered since that would confront the patient's both problems. Nonetheless, the modified anatomy that we have already mentioned makes THA a demanding and challenging technique with failure rates ranging from 14% to 29% in ankylosed hip [13–15]. Tsakotos et al. successfully treated a midshaft femoral fracture in a 57-year-old lady with coexisting congenital dysplasia and ankylosis of the ipsilateral hip, by performing total hip arthroplasty with the use of a long stem that acted as an intramedullary nail [11]. However, in our case, the patient suffered from bilateral CHD, so THA would indicate a second operation in the future for the reconstruction of the contralateral hip in order to confront the consequent leg length discrepancy. Additionally, our patient was 74 years old, ambulatory with a walking stick, capable of all self-care, and contented with her previous walking status. Furthermore, the patient did not experience severe pain before the injury, neither nocturnal pain, and she was not receiving analgetics in a regular basis. The preexisting limp did not affect her everyday activities and her quality of life. As a result, the patient had never sought treatment before, and she had never been in a list for surgery. However, the option of a total hip arthroplasty with subtrochanteric shortening was discussed with her but was rejected as she did not accept the possible risks of such an operation.

By contrast, open internal fixation using this specially designed periprosthetic femur plate proved to be a successful method of stabilization after open anatomical reduction. The specific plate fits anatomically to the lateral wall of the femur, extends to the greater trochanter, and facilitates the placement of multiple bicortical locking screws of various diameter sizes in different directions proximally and distally, adding stability to the fixation.
